# Correlation of levels of lactic acid and glucose in cerebrospinal fluid of cerebral hemorrhage patients with the occurrence of postoperative intracranial infection and clinical prognosis

**DOI:** 10.5937/jomb0-44058

**Published:** 2024-01-25

**Authors:** Lei Zhang, Yan Zhang, Xiaotian Wang, Yun Zhao

**Affiliations:** 1 Dongying Peoples Hospital, Department of Emergency Critical Care Medicine, Dongying, China; 2 Dongying Peoples Hospital, Administration Department of Nosocomial Infection Dongying, China

**Keywords:** cerebral haemorrhage, lactic acid, glucose, postoperative intracranial infection, clinical prognosis, correlation, cerebralno krvarenje, mlečna kiselina, glukoza, postoperativna intrakranijalna infekcija, klinička prognoza, korelacija

## Abstract

**Background:**

Cerebral haemorrhage is a critical condition that often requires surgical treatment, and postoperative intracranial infection can significantly impact patient outcomes. The aim of the study was to examine the relationship between the levels of lactic acid and glucose in cerebrospinal fluid (CSF) of patients with cerebral haemorrhage and their postoperative intracranial infection and clinical prognosis.

**Methods:**

The study selected the clinical data of 324 patients with cerebral haemorrhage who underwent surgical treatment in our hospital from March 2020 to March 2022 for retrospective analysis and divided these patients into the intracranial infection group (Group A, n=22, leukocyte values in CSF>5×106/L) and the non-intracranial infection group (Group B, n=302, leukocyte values in CSF 5×106/L) according to the occurrence of postoperative intracranial infection in patients to detect the levels of lactic acid and glucose in CSF at different times in the two groups. Pearson method was adopted to analyze the correlation of the levels of lactic acid and glucose in CSF of patients with intracranial infection, and the Glasgow Outcome Scale (GOS) was used to assess the clinical prognosis of patients. According to their scores, these patients were divided into the good prognosis group (GPG, scores of 4-5 points, n=178) and the poor prognosis group (PPG, scores of 1-3 points, n=146). The levels of lactic acid and glucose in the CSF of patients in the two groups were measured, and the Pearson method was adopted to analyze the relationship between these levels and clinical prognosis.

## Abstract (sr)

**Uvod:**

Cerebralno krvarenje je kritično stanje koje često zahteva hirurško lečenje, a postoperativna intrakranijalna infekcija može značajno da utiče na ishod. Cilj studije je bio da se analizira korelacija nivoa mlečne kiseline i glukoze u cerebrospinalnoj tečnosti (CSF) pacijenata sa cerebralnim krvarenjem sa pojavom postoperativne intrakranijalne infekcije i kliničkom prognozom.

**Metode:**

U studiji su selektovani klinički podaci 324 pacijenta sa cerebralnom hemoragijom koji su podvrgnuti hirurškom lečenju u našoj bolnici od marta 2020. do marta 2022. godine radi retrospektivne analize. Pacijenti su podeljeni u grupu intrakranijalne infekcije (Grupa A, n=22, vrednosti leukocita u CSF>5×106/L) i grupu bez intra-kranijalne infekcije (Grupa B, n=302, vrednosti leukocita u CSF 5×106/L) prema pojavi postoperativne intrakranijalne infekcije kod pacijenata. U obe grupe su mereni nivoi mlečne kiseline i glukoze u CSF u različitim vremenima. Korišćena je Personova metoda za analizu korelacije nivoa mlečne kiseline i glukoze u CSF kod pacijenata sa intra-kranijalnom infekcijom, a za procenu kliničke prognoze pacijenata je korišćena Glasgow Outcome Scale (GOS). Prema njihovim rezultatima, pacijenti su podeljeni u dve grupe: grupu sa dobrim ishodom (GPG, 4-5 poena, n=178) i grupu sa lošim ishodom (PPG, 1-3 poena, n=146). Mereni su nivoi mlečne kiseline i glukoze u CSF kod pacijenata u obe grupe, a Personova metoda je korišćena za analizu veze između tih nivoa i kliničke prognose.

**Rezultati:**

U poređenju sa Grupom B, Grupa A je imala značajno više nivoe mlečne kiseline u CSF pacijenata u vreme T1, T2 i T3 (P<0,001), i primetno niže nivoe glukoze (P<0.001). Pacijenti u PPG grupi su imali primetno više nivoe mlečne kiseline u CSF u vreme T1, T2 i T3 (P<0,001) i jasno niže nivoe glukoze u poređenju sa GPG (P<0,001). Rezultati Personove metode su pokazali da su nivoi mlečne kiseline u CSF pacijenata pozitivno korelisani sa vrednostima leukocita, ali negativno korelisani sa nivoima glukoze (P<0,05). Nivoi mlečne kiseline u CSF su u negativnoj korelaciji sa rezultatima GOS skale, ali u pozitivnoj korelaciji sa nivoima glukoze (P<0,05).

**Zaključak:**

Nivoi mlečne kiseline i glukoze u cerebrospinalnoj tečnosti (CSF) kod pacijenata sa cerebralnim krvarenjem su povezani sa postoperativnom intrakranijalnom infekcijom i kliničkom prognozom, a detekcija gore navedenih pokazatelja pomaže lekarima da bolje razumeju stanje pacijenata, pružajući naučnu osnovu za formulisanje kliničkih terapijskih planova.

## Introduction

Cerebral haemorrhage is a primary disease that causes adult disability with high mortality. The main mechanism of early brain injury after cerebral haemorrhage is that blood accumulates locally after cerebral haemorrhage to form a hematoma, which can directly stimulate and compress the surrounding brain tissues, and cerebral haemorrhage often causes various symptoms of neurologic impairment, such as limb paralysis, language disorder and disturbance of consciousness [1]. Surgery is still the first choice for treating cerebral haemorrhage, whose therapeutic objective is reducing the compression of hematoma on patients' brain tissues. At the same time, the toxic substances such as batroxobin and 5-hydroxytryptamine produced after haemorrhage lead to aggravation of cerebral oedema, and clearing the hematoma can alleviate the secondary pathological and physiological vicious cycles and effectively control the vasogenic and cytogenic brain oedema [2]. However, this surgical treatment and retention of the postoperative drainage tube may lead to intracranial infection. In contrast, intracranial infection, one of the major factors causing a poor prognosis, can aggravate the neurological impairment of patients, prolong hospitalization time and increase the mortality of patients [3] [4]. Bacteriological detection of cerebrospinal fluid (CSF) is the »golden standard« for clinical diagnosis of intracranial infection, but it is somewhat difficult to diagnose early intracranial infection because of longer culture time and a lower rate of positive cultivation [5].

How to develop more scientific and objective diagnostic indicators becomes a critical subject facing clinicians. Studies have pointed out [6] that lactic acid, a product of anaerobic glycolysis of tissues, has notably higher levels of CSF compared with serum levels in the occurrence of intracranial bacterial infection. When the blood perfusion of brain tissues decreases, the oxygen supply of the brain will decrease, and the anaerobic glycolysis of brain tissues and lactic acid levels will increase [7]. In addition, studies have also found [8] [9] that there is a correlation between glucose content in CSF and central nervous system (CNS) infection, and the permeability of the CSF barriers is related to the extent of glucose glycolysis in CSF. Moreover, when infection occurs in CNS, glucose content in CSF decreases under the enzymolysis released by pathogens and damaged cells. There is little literature at home and abroad to confirm the correlation of levels of lactic acid and glucose in CSF of cerebral haemorrhage patients with postoperative intracranial infection and clinical prognosis. By conducting clinical trials, this study adopts levels of lactic acid and glucose in CSF to predict postoperative intracranial infection and prognosis of patients with cerebral haemorrhage, thereby further accurately assessing the disease progression and prognosis of such patients, which is reported as follows.

## Materials and methods

### General information

The study selected the clinical data of 324 patients with cerebral haemorrhage who underwent surgical treatment in our hospital from March 2020 to March 2022 for retrospective analysis, and the study has been reviewed and approved by the ethical committee of our hospital, conforming to the Declaration of Helsinki (2013) [10]. All family members of patients were informed about the purpose and process of this pilot study and signed informed consent.

### Inclusion and exclusion criteria

Inclusion criteria: (1) Patients met the diagnostic criteria of cerebral haemorrhage according to the Chinese Stroke Association Guidelines for Clinical Management of Cerebrovascular Disorders [11] and were diagnosed through clinical, magnetic resonance imaging (MRI) and computed tomography (CT) examinations. (2) Patients received treatment within 12 h at first morbidity [3]. Patients had no disease affecting the physiological status of cerebral vessels, such as arteriosclerosis and cerebrovascular malformation, and no other neurological disease before the injury. 

*Exclusion criteria*: (1) Patients with severe dysfunctions in the kidney, heart, liver and other organs; (2) patients with previous diseases such as brain tumour and cerebral infarction; (3) patients with haematological diseases; and (4) patients who were in pregnancy or lactation.

### Methods

The first (T1) sample, derived from the cerebral ventricles, was immediately detected after ventricular drainage. The supernatant was extracted through centrifugation and precipitation before detection. Samples with hemolysis or more than 5% of interfering redundant erythrocytes were subsampled. Lactic acid levels were measured using the enzyme kinetics method with an automatic blood gas electrolyte analyzer, the Hitachi 7600 automatic biochemical analyzer (manufacturer: Shanghai Huanxi Medical Instruments Co., Ltd.), and matching reagents. The glucose levels in patient's cerebrospinal fluid (CSF) were measured using the glucose oxidase method.

The second (T2) sample was detected before removing the ventricular drainage tube, and the detection methods and procedures were the same as above.

The third (T3) sample was detected postoperatively, lumbar puncture was adopted to obtain CSF from patients, the matching reagents of Gem Premier 3500 (Instrumentation Laboratory, US) were used to detect lactic acid levels, and the methods and procedures of glucose detection were the same as above. The normal Lactic acid levels in CSF range from 0.999 to 2.775 mmol/L, and normal glucose levels in CSF range from 2.5 to 4.4 mmol/L in humans.

### Statistical analysis

The experimental data were all processed by Statistical Product and Service Solutions (SPSS) 26.0 software (SPSS Inc., Chicago, IL, USA) for statistical analysis, PASS software was adopted to calculate the study's sample size, and GraphPad Prism 7 (Graph-Pad Software, San Diego, USA) was used for image drawing. The enumeration data and the measurement data were detected by x^2^ and t-test, indicated by (n (%)) and (x̄ ± standard deviation (Sd)), respectively. The Pearson method was used to analyze the correlation of each indicator with GOS scores and leukocyte values, and the difference was considered statistically significant when P<0.05.

## Results

### Comparison of clinical data of patients

There was no notable difference in clinical data such as age, BMI values and residence between the intracranial infection group (Group A) and the nonintracranial infection group (Group B) (P>0.05), with comparability, as shown in Table 1. There was no significant difference in age, BMI values and residence between the good prognosis group (GPG) and the poor prognosis group (PPG) (P>0.05), with comparability, as shown in Table 2.

**Table 1:** Comparison of clinical data between Group A and Group B.

Projects	Group A (n=22)	Group B (n=302)	x^2^/t	P
Gender			0.774	0.379
Male	15 (68.18)	231 (76.49)		
Female	7 (31.82)	71 (23.51)		
Age (x̄±s, years old)	62.05±11.45	58.17±10.20	1.874	0.075
BMI values (x̄±s, kg/m^2^)	21.20±1.48	21.03±1.50	0.032	0.975
Time from injury to admission (x̄±s, h)	4.41±1.05	4.69±1.10	0.499	0.623
Causes of injury			0.001	0.981
Spontaneous cerebral haemorrhage	20 (90.91)	275 (91.06)		
Cerebral haemorrhage caused by brain trauma	2 (9.09)	27 (8.94)		
Bleeding parts			2.442	0.486
Putaminal regions	14 (63.64)	231 (76.49)		
Thalamus	4 (18.18)	43 (14.24)		
Cerebral ventricles	2 (9.09)	12 (3.97)		
Others	2 (9.09)	16 (5.30)		
Residence			0.454	0.500
City	9 (40.91)	146 (48.34)		
Countryside	13 (59.09)	156 (51.66)		

**Table 2:** Comparison of clinical data between the GPG and the PPG.

Projects	GPG (n=178)	PPG (n=146)	x^2^/t	P
Gender			0.008	0.927
Male	96 (53.93)	78 (53.42)		
Female	82 (46.07)	68 (46.58)		
Age (x̄±s, years old)	58.37±10.55	57.87±10.71	0.275	0.784
BMI values (x̄±s, kg/m^2^)	21.04±1.55	21.23±1.61	0.883	0.379
Time from injury to admission (x̄±s, h)	4.48±1.16	4.47±1.18	0.307	0.759
Causes of injury			0.654	0.419
Spontaneous cerebral haemorrhage	160 (89.89)	135 (92.47)		
Cerebral haemorrhage caused by brain trauma	18 (10.11)	11 (7.53)		
Bleeding parts			5.120	0.163
Putaminal regions	118 (66.29)	97 (66.44)		
Thalamus	42 (23.60)	28 (19.18)		
Cerebral ventricles	6 (3.37)	13 (8.90)		
Others	12 (6.74)	8 (5.48)		
Residence			0.003	0.955
City	92 (51.69)	75 (51.37)		
Countryside	86 (48.31)	71 (48.63)		

### Comparison of levels of lactic acid and glucose in CSF of patients at different times between Group A and Group B

At T1, T2, and T3, patients in Group A had significantly higher lactic acid levels in CSF (P<0.001), whereas they had notably lower glucose levels than those in Group B (P<0.001), as shown in Table 3 and Table 4.

**Table 3:** Comparison of lactic acid levels in CSF of patients at different times between the two groups.

Groups	n	T1	T2	T3
Group A	22	5.40±0.54	5.20±0.64	5.25±0.63
Group B	302	1.47±0.23	1.48±0.23	1.46±0.22
t		31.080	24.944	26.962
P		<0.001	<0.001	<0.001

**Table 4:** Comparison of glucose levels in CSF of patients at different times between the two groups.

Groups	n	T1	T2	T3
Group A	22	2.47±0.44	2.56±0.36	2.45±0.37
Group B	302	3.66±0.56	3.64±0.56	3.64±0.52
t		5.825	7.851	9.000
P		<0.001	<0.001	<0.001

### Comparison of levels of lactic acid and glucose in CSF of patients between the GPG and the PPG

At T1, T2, and T3, patients in the PPG had notably higher lactic acid levels in CSF (P<0.001), whereas they had notably lower glucose levels than those in the GPG (P<0.001), as shown in Table 5 and Table 6.

**Table 5:** Comparison of lactic acid levels in CSF of patients at different times between the two groups.

Groups	n	T1	T2	T3
GPG	178	2.59±0.23	2.59±0.21	2.60±0.25
PPG	146	4.03±0.73	3.95±0.68	4.00±0.74
t		23.688	23.988	22.418
P		<0.001	<0.001	<0.001

**Table 6:** Comparison of glucose levels in CSF of patients at different times between the two groups.

Groups	n	T1	T2	T3
GPG	178	3.96±0.41	3.89±0.40	3.95±0.43
PPG	146	3.12±0.37	3.08±0.35	3.11±0.36
t		17.954	19.842	17.606
P		<0.001	<0.001	<0.001

### Correlation of levels of lactic acid and glucose in CSF with intracranial infection

Results of Pearson showed that lactic acid levels in CSF of patients were positively correlated with leukocyte values but negatively correlated with glucose levels (P<0.05), as shown in Table 7.

**Table 7:** Correlation of levels of lactic acid and glucose in CSF of patients with leukocyte values.

Indexes	Leukocyte values	
	r	P
Lactic acid	0.400	<0.05
Glucose	-0.973	<0.05

### Correlation of levels of lactic acid and glucose in CSF with GOS scores

Results of Pearson showed that lactic acid levels in CSF of patients were negatively correlated with GOS scores but positively correlated with glucose levels (P<0.05), as shown in Table 8.

**Table 8:** Correlation of levels of lactic acid and glucose in CSF of patients with GOS scores.

Indexes	GOS scores	
	r	P
Lactic acid	-0.848	<0.05
Glucose	0.921	<0.05

## Discussion

Cerebral haemorrhage, which can have multiple underlying causes, is predominantly attributed to the rupture of hypertensive blood vessels with arteriolosclerosis [12]. It is a frequent neurosurgical emergency that leads to brain damage, including localized compression from the hematoma, injuries caused by releasing inflammatory factors and free radicals, and delayed cerebral injury characterized by brain cell oedema and metabolic dysfunction [13]. Patients with cerebral haemorrhage often experience complex and severe injuries, with rapidly changing disease conditions and challenging treatment scenarios. Despite the advancements in clinical therapeutic techniques and the implementation of aseptic and antibacterial surgical practices, postoperative intracranial infection remains a concern. This underscores the need for clinicians to establish a reliable prognostic evaluation system to guide treatment decisions during the management of these patients [14] [15].

Lactic acid is a metabolic end product of glycolysis, and the concentration of lactic acid in CSF can reflect the glycolytic metabolism of CNS. When infections occur in patients [16], leukocytes will increase the anaerobic metabolism of glucose, thereby producing lactic acid and reducing PH values. The lactic acid in CSF is not affected by blood lactate levels, so the concentration in CSF reflects the extent of CNS infection in the body to some extent [17] [18]. Some scholars have found [19] that when the body is infected, capillary endothelial damage and oedema of local tissues cause the decrease of microvessel density in tissues. Accumulation of lactic acid and the subsequent increase in its concentration occur due to metabolic dysfunction in local tissues and the impaired local clearance of lactic acid. Therefore, lactic acid levels are considered to be positively correlated with body infection. Through examining lactic acid levels in CSF between Group A and Group B after surgery, the study found that patients in Group A had overtly higher lactic acid levels in CSF than those in Group B (P<0.001), predicting that patients with postoperative infection after traumatic brain injury have ischemia and hypoxia of local nerve cells to cause a massive release of lactic dioxygenases from nerve cells [20] [21] and an increase of the lactic acid concentration in CSF. It was further found that lactic acid levels in patients' CSF were positively correlated with leukocyte values (r=0.400, P<0.05), indicating that the detection of lactic acid levels in patients with cerebral haemorrhage can effectively predict whether patients have intracranial infection postoperatively.

Glucose is an important energy source for nerve cells, while bacteria also produce energy for their use by decomposing glucose [22] [23]. When a bacterial infection occurs, bacteria decompose glucose in CSF, leading to lower glucose levels in the CSF. Therefore, it was found that patients in Group A had overtly lower glucose levels in CSF than those in Group B, indicating that glucose is involved in developing and progressing postoperative intracranial infection in patients. The study further found that glucose levels in CSF were negatively correlated with leucocyte values (r=-0.973, P<0.05), indicating that the detection of glucose levels in CSF of patients can effectively predict whether patients have intracranial infection postoperatively. GOS score is a common scoring standard in neurosurgery, which has some auxiliary values in evaluating the prognosis of patients with conscious changes [24]. This study found that patients in the PPG had notably higher lactic acid levels in CSF and significantly lower glucose levels than those in the GPG (P<0.001). Results of Pearson showed that lactic acid levels in CSF of patients were negatively correlated with GOS scores but positively correlated with glucose levels (P<0.05), indicating that the detection of levels of lactic acid and glucose in CSF of patients with cerebral haemorrhage can effectively predict clinical prognosis.

The study has several limitations that should be considered when interpreting the findings. Firstly, the study relied on a retrospective analysis of clinical data, which introduces inherent limitations such as potential biases and incomplete data. This retrospective approach may limit the ability to establish a causal relationship between the levels of lactic acid and glucose in cerebrospinal fluid (CSF) and postoperative intracranial infection and clinical prognosis. Secondly, the sample size of the intracranial infection group was relatively small, which may limit the generalizability of the results. Additionally, the study focused specifically on patients with cerebral haemorrhage who underwent surgical treatment, which restricts the applicability of the findings to other types of brain injuries or patients who received non-surgical interventions. Furthermore, the study did not account for other potential factors that could influence the occurrence of postoperative intracranial infection and clinical prognosis, such as comorbidities or specific treatment protocols. Finally, the study solely examined the correlation between lactic acid and glucose levels in CSF and clinical outcomes without delving into the underlying mechanisms or pathways involved. Despite these limitations, the study provides valuable insights into the potential association between lactic acid, glucose, and clinical outcomes in cerebral haemorrhage patients, but further research is needed to validate the findings and explore the underlying mechanisms in more depth. However, there are also some deficiencies in this trial. For example, the research subjects are mainly adults without including children, and other CSF, such as uric acid and lactic dehydrogenase, are not widely studied, which may have greater potential values for intracranial infection. Despite some limitations, this study presents valuable research conclusions, providing a reliable basis for the clinical treatment of patients with cerebral haemorrhage.

## Dodatak

### Conflict of interest statement

All the authors declare that they have no conflict of interest in this work.
